# Tislelizumab plus tyrosine kinase inhibitors with TACE improves survival in unresectable hepatocellular carcinoma with clinical predictors and manageable safety

**DOI:** 10.3389/fimmu.2025.1664519

**Published:** 2025-09-29

**Authors:** Fengliang Wang, Zhenxue Cao, Chunpeng Yu, Jian Li, Qun Li, Shuai Chang, Shuo Zhang, Song Wang

**Affiliations:** ^1^ Interventional Medical Center, The Affiliated Hospital of Qingdao University, Qingdao, Shandong, China; ^2^ Department of Internal Medicine, Qingdao Cardiovascular Hospital, Qingdao, Shandong, China

**Keywords:** hepatocellular carcinoma, tislelizumab, tyrosine kinase inhibitors (TKIs), transarterial chemoembolization (TACE), survival analysis

## Abstract

**Background & Aims:**

The survival benefit of adding transarterial chemoembolization (TACE) to systemic therapy (tislelizumab plus tyrosine kinase inhibitors [TKIs]) for unresectable hepatocellular carcinoma (HCC) requires validation. This retrospective study compared the efficacy and safety of tislelizumab-TKIs with or without TACE and identified clinical predictors of benefit.

**Methods:**

This retrospective analysis included 283 unresectable HCC patients: systemic therapy alone (STG, n=98; tislelizumab plus TKIs) versus combination therapy (CTG, n=185; tislelizumab plus TKIs and TACE). Primary endpoints were overall survival (OS) and progression-free survival (PFS), analyzed by Cox regression. Propensity score matching (PSM) was used to reduce baseline differences between the two groups.

**Results:**

After PSM, CTG significantly improved median OS (22.5 [95% confidence interval (CI): 19.0–34.4] *vs*. 14.0 [12.1–18.6] months; hazard ratio (HR) 0.53, p<0.001) and PFS (14.6 [12.1–19.1] *vs*. 9.5 [7.8–12.5] months; HR 0.59, p<0.001) versus STG. Multivariate analysis identified independent predictors of poor OS: age <60 years, extrahepatic spread, portal vein thrombus, alpha-fetoprotein (AFP) ≥400 ng/mL, and elevated gamma-glutamyl transferase (GGT). Subgroups with maximal CTG benefit included patients aged ≥60 years, no extrahepatic spread, AFP <400 ng/mL, and normal GGT. CTG had higher all-grade adverse events (79.6% *vs*. 67.0%, p=0.021) and grade ≥3 events (23.5% *vs*. 14.1%, p=0.038), primarily manageable liver toxicity and hematological abnormalities.

**Conclusion:**

Combining TACE with tislelizumab-TKIs significantly improves survival over systemic therapy alone in unresectable HCC, with maximal benefit observed in patients aged ≥60 years, without extrahepatic spread, with AFP <400 ng/mL, or normal GGT, despite increased manageable toxicity.

## Highlights

- Tislelizumab + TKIs + TACE significantly improved median OS (22.5 *vs*. 14.0 months) and PFS (14.6 *vs*. 9.5 months) over systemic therapy alone in unresectable HCC.
- Maximal OS benefit from triple therapy occurred in patients aged ≥60 years, without extrahepatic spread, AFP <400 ng/mL, or normal GGT levels.
- Triple therapy increased all-grade AEs (79.6% *vs*. 67.0%) and grade ≥3 AEs (23.5% *vs*. 14.1%), but had comparable treatment discontinuation rates, supporting clinical feasibility.
- Abbreviations
- TACE transarterial chemoembolization; HCC hepatocellular carcinoma; TKIs tyrosine kinase inhibitors; STG systemic therapy group; OS overall survival; PFS progression-free survival; PSM propensity score matching; CI confidence interval; HR hazard ratio; AFP alpha-fetoprotein; GGT gamma-glutamyl transferase; CTG combination therapy group; BCLC Barcelona clinic liver cancer; ICIs immune checkpoint inhibitors; DEB-TACE drug-eluting bead transarterial chemoembolization; PD-1 programmed cell death protein 1; CT computed tomography; MRI magnetic resonance imaging; IQR interquartile range

## Introduction

Hepatocellular carcinoma (HCC) remains a formidable global health burden, accounting for over 900,000 annual diagnoses and ranking as the third leading cause of cancer-related mortality (1–3). Despite therapeutic advancements, unresectable advanced-stage HCC continues to portend a dismal prognosis (4, 5). First-line systemic therapies combining immune checkpoint inhibitors (ICIs) and tyrosine kinase inhibitors (TKIs), such as atezolizumab-bevacizumab, have improved outcomes but demonstrate suboptimal efficacy in real-world populations, particularly among patients with high intrahepatic tumor burden, vascular invasion, or compromised liver function (6–9). Concurrently, transarterial chemoembolization (TACE), the standard for intermediate-stage HCC, achieves localized tumor control through ischemic necrosis and intra-arterial chemotherapy but fails to address systemic progression (10–12). This therapeutic dichotomy underscores the urgent need for synergistic strategies integrating locoregional and systemic modalities.

Emerging preclinical evidence supports the biological rationale for combining TACE with immunotherapy and TKIs (13–15). TACE may potentiate anti-programmed cell death protein-1 (PD-1) efficacy by releasing tumor-associated antigens and modulating the immunosuppressive microenvironment via hypoxia-inducible factor downregulation, while TKIs could counteract post-TACE VEGF-driven angiogenesis (16–21). However, clinical validation remains limited to small single-arm studies lacking real-world evidence on the synergistic potential of locoregional-systemic combination therapy, with implications for refining clinical decision-making and guiding future prospective trial designs. This study aimed to compare the efficacy and safety of tislelizumab-TKIs with or without TACE and identified patient subgroups benefiting from combined modality therapy.

## Methods

### Study design and participants

**Figure 1:**
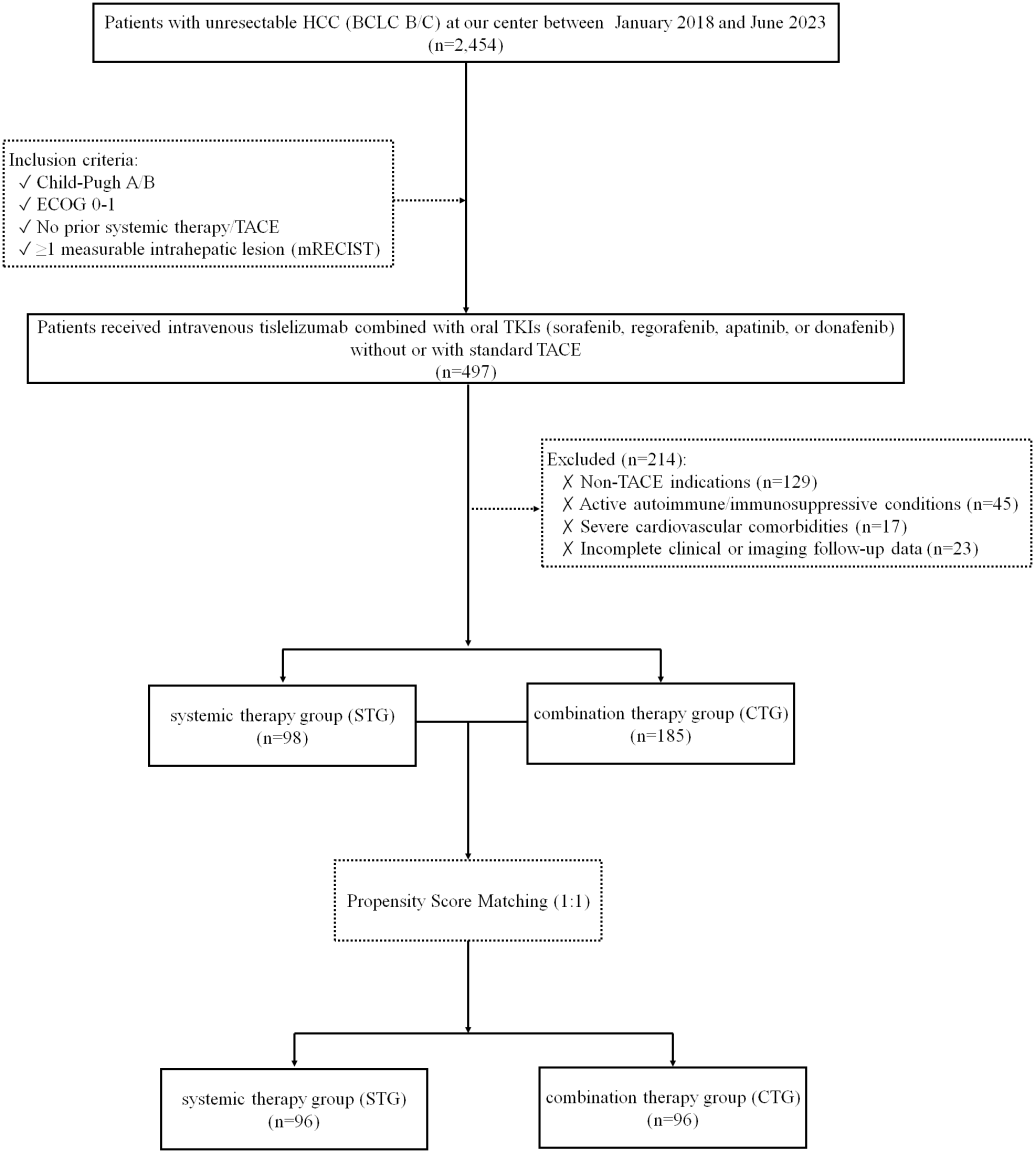
Study flowchart.

This retrospective cohort study enrolled consecutive patients diagnosed with unresectable HCC, classified as Barcelona Clinic Liver Cancer (BCLC) stage B or C, at a single tertiary center between January 2018 and June 2023. Inclusion criteria were Child-Pugh class A or B liver function, Eastern Cooperative Oncology Group (ECOG) performance status 0–1, no prior exposure to systemic therapy or TACE, and the presence of at least one measurable intrahepatic lesion according to modified Response Evaluation Criteria in Solid Tumors (mRECIST) (22). Exclusion criteria included non-TACE indications, active autoimmune diseases or ongoing immunosuppressive therapy, incomplete clinical or imaging follow-up data, and severe cardiovascular comorbidities such as uncontrolled hypertension or New York Heart Association class III/IV heart failure. Patients were divided into either the systemic therapy group (STG) or the combination therapy group (CTG), based on whether they received treatment without or with TACE. The grouping of patients (STG *vs*. CTG) was primarily determined by whether they met the clinical criteria for TACE treatment (e.g., liver function, tumor burden, portal vein invasion status, etc.), rather than random assignment. The patient screening flowchart is presented in **Figure 1**.

### TACE procedure

Patients in the CTG received standard TACE using a lipiodol-based chemotherapeutic emulsion (containing pirarubicin, 30–50 mg; lobaplatin, 30–50 mg; and lipiodol, 2–15 ml), followed by embolization with microspheres and gelatin sponge particles (100–300 μm) (23). The chemotherapeutic agent dosage was adjusted according to tumor size and liver function. Repeat TACE procedures were performed on an on-demand basis upon imaging evidence (contrast-enhanced CT or MRI) of active tumor or intrahepatic recurrence. All TACE procedures were conducted by interventional radiologists at our center with at least 5 years of experience. Under DSA guidance, selective catheterization of tumor-feeding arteries was performed, followed by embolization until stasis of contrast flow. Repeat TACE sessions were administered for residual or recurrent lesions, with intervals ≥4 weeks between procedures. Post-procedure management included hydration, analgesics, and monitoring for embolization-related complications.

### Molecular targeted agents and tislelizumab administration

In the STG, patients received intravenous tislelizumab (200 mg every 3 weeks) combined with oral TKIs: sorafenib (400 mg twice daily), lenvatinib (8 mg/day for body weight <60 kg or 12 mg/day for ≥60 kg), regorafenib (80, 120 or 160 mg once daily for 3 weeks followed by 1 week off), apatinib (500 or 750 mg once daily), or donafenib (200 mg twice daily); with TKI dose reductions permitted for grade ≥3 adverse events (AEs). The CTG group initiated the same systemic regimen (tislelizumab plus TKIs) within 7 days after the first TACE session, with subsequent TACE cycles synchronized to systemic therapy and temporary TKI interruption (≤7 days) during TACE procedures. Treatment protocols included dose adjustments for TKIs (50% reduction for grade 3 AEs and discontinuation for grade 4 events) and permanent discontinuation of tislelizumab for unresolved grade ≥3 immune-related AEs despite corticosteroid therapy. Systemic therapy was continued until disease progression, intolerable toxicity, or patient withdrawal.

### Assessments

Tumor response was evaluated using contrast-enhanced CT or MRI at baseline and every 8 (± 1) weeks thereafter. The radiologists involved in assessing tumor response were not formally blinded to the treatment groups. All radiographic assessments were conducted according to the standardized mRECIST criteria. The scans were reviewed independently by two experienced radiologists, with any discrepancies resolved by a third senior radiologist to reach a consensus. Clinical and laboratory variables collected included: extrahepatic spread, portal vein tumor thrombus, serum alpha-fetoprotein (AFP), and gamma-glutamyl transferase (GGT) levels. Safety monitoring adhered to Common Terminology Criteria for Adverse Events (CTCAE) version 5.0, with specific documentation of TACE-related complications.

### Statistical analysis

Overall survival (OS) was defined as the time from treatment initiation to death from any cause. Progression-free survival (PFS) was measured as the time to radiologic progression or death. Survival curves were generated using the Kaplan-Meier method and compared via log-rank test. Univariate and Multivariate Cox regression analyses were performed for all baseline variables, including sex, age, surgical resection, TKI agents (sorafenib, regorafenib, apatinib, donafenib; reference: lenvatinib), TACE, etiology, extrahepatic spread, tumor diameter, tumor number, portal vein tumor thrombus, Child-Pugh class, BCLC stage, MELD score, AFP, platelet count, prothrombin time (PT), international normalized ratio (INR), albumin, serum creatinine (Scr), GGT, cholinesterase, total bilirubin, hemoglobin, and lymphocyte count. The cut-off values were based on either established clinical standards (as for AFP) or ROC-derived values (as for GGT). To minimize the potential influence of confounding factors and reduce selection bias affecting OS and PFS, baseline patient characteristics between the two groups were matched using 1:1 propensity score matching (PSM). The matching variables included sex, age, surgical resection, type of TKIs, hepatitis status, extrahepatic spread, tumor diameter, tumor number, BCLC stage, portal vein tumor thrombus, Vp type, Child-Pugh class, MELD score, AFP level, and platelet count. Treatment effect heterogeneity was evaluated using likelihood ratio tests. All analyses were conducted using R software (version 4.2.2) and SPSS (version 26.0). A two-sided P-value <0.05 defined statistical significance.

## Results

### Baseline characteristics

This retrospective analysis included 283 consecutive patients with unresectable HCC treated between January 2018 and June 2023, comprising 185 patients receiving combination therapy (tislelizumab + TKIs + TACE) and 98 receiving systemic therapy alone (tislelizumab + TKIs). Baseline characteristics were well-balanced between groups (p >0.05 for all comparisons): The cohort had a median age of 58 years (interquartile range [IQR] 52–65), with male predominance (85.5%) and hepatitis B as the primary etiology (85.9%). Key clinical features including extrahepatic spread (36.2% *vs*. 48.0%, p=0.055), portal vein tumor thrombus (34.1% *vs*. 34.7%, p=0.914), AFP ≥400 ng/mL (33.0% *vs*. 27.6%, p=0.348), tumor diameter ≥5cm (56.2% *vs*. 52.0%, p=0.502), and BCLC stage distribution (Stage B: 38.9% *vs*. 37.8%; Stage C: 61.1% *vs*. 62.2%, p=0.848) showed no statistically significant differences. No significant baseline differences were observed between the two groups (**Table 1**). After PSM, each group contained 96 individuals, and the baseline differences between them were significantly reduced, as detailed in **Supplementary Table 1**.

**Table 1:** Patient baseline characteristics before PSM.

Characteristics, n (%)	CTG(n=185)	STG(n=98)	P-value
Sex			0.127
Male	156 (84.3)	89 (90.8)	
Female	29 (15.7)	9 (9.2)	
Age, (years)			0.301
<60	75 (40.5)	46 (46.9)	
≥60	110 (59.5)	52 (53.1)	
Age, median (IQR)	61 (54, 67)	60 (54, 65.8)	0.135
Surgical resection			0.910
Yes	78 (42.2)	42 (42.9)	
No	107 (57.8)	56 (57.1)	
TKIs			0.887
Lenvatinib	99 (53.5)	49 (50)	
Sorafenib	11 (5.9)	5 (5.1)	
Regorafenib	65 (35.1)	39 (39.8)	
Apatinib	6 (3.2)	2 (2)	
Donafenib	4 (2.2)	3 (3.1)	
Hepatitis B			0.259
Yes	162 (87.6)	81 (82.7)	
No	23 (12.4)	17 (17.3)	
Extrahepatic spread			0.055
Yes	67 (36.2)	47 (48)	
No	118 (63.8)	51 (52)	
Tumor diameter			0.502
<5cm	81 (43.8)	47 (48)	
≥5cm	104 (56.2)	51 (52)	
Tumor diameter cm, median (IQR)	5.7 (3, 8.1)	5.1 (3.1, 8.0)	0.761
Tumor number			0.269
<3	56 (30.3)	36 (36.7)	
≥3	129 (69.7)	62 (63.3)	
BCLC stage			0.848
B	72 (38.9)	37 (37.8)	
C	113 (61.1)	61 (62.2)	
Portal vein tumor thrombus			0.914
Yes	63 (34.1)	34 (34.7)	
NO	122 (65.9)	64 (65.3)	
Vp type			0.924
I	2 (3.2)	1 (2.9)	
II	42 (66.7)	24 (70.6)	
III	19 (30.2)	9 (26.5)	
Child-Pugh class			0.715
A	136 (73.5)	74 (75.5)	
B	49 (26.5)	24 (24.5)	
MELD score			0.281
<18	69 (37.3)	43 (43.9)	
≥18,	116 (62.7)	55 (56.1)	
AFP (ng/ml)			0.348
<400	124 (67)	71 (72.4)	
≥400	61 (33)	27 (27.6)	
Platelet (10^9/L)			0.448
<100	67 (36.2)	40 (40.8)	
≥100	118 (63.8)	58 (59.2)	
PT(sec), median (IQR)	13.2 (12.1, 14.2)	13.15 (12.0, 14.2)	0.919
INR, median (IQR)	1.12 (1.0, 1.2)	1.105 (1.1, 1.2)	0.537
Albumin(g/l), median (IQR)	38.7 (35.3, 42.6)	39.75 (34.4, 42.9)	0.588
Serum creatinine(mg/dL), median (IQR)	0.90045 (0.8, 1.0)	0.88801 (0.8, 1.0)	0.571
GGT(U/L), median (IQR)	53.6 (30, 123.9)	61.7 (30.2, 136.6)	0.623
Cholinesterase(U/L), median (IQR)	6349 (4903.0, 8202.8)	5874.5 (4289.2, 8376.2)	0.319
Total bilirubin(mg/dl), median (IQR)	1.3216 (0.9, 1.8)	1.2459 (0.9, 1.7)	0.375
Hemoglobin(g/l), median (IQR)	140 (126, 151)	140 (119.3, 154.0)	0.758
Lymphocyte count(10^9/L), median (IQR)	1.27 (0.8, 1.7)	1.255 (0.9, 1.8)	0.917

Unless otherwise indicated, data are the number of patients or median (interquartile range), with percentages in parentheses; A P-value <0.05 was considered to indicate statistical significance. PSM, propensity score matching; CTG, combination therapy group; STG, systemic therapy group; IQR, interquartile range; TKIs, tyrosine kinase inhibitors; BCLC, barcelona clinic liver cancer; MELD, Model for end-stage liver disease; AFP, alpha-fetoprotein; PT, prothrombin time; INR, international normalized ratio; GGT, gamma-glutamyl transferase.

### Survival outcomes

**Figure 2:**
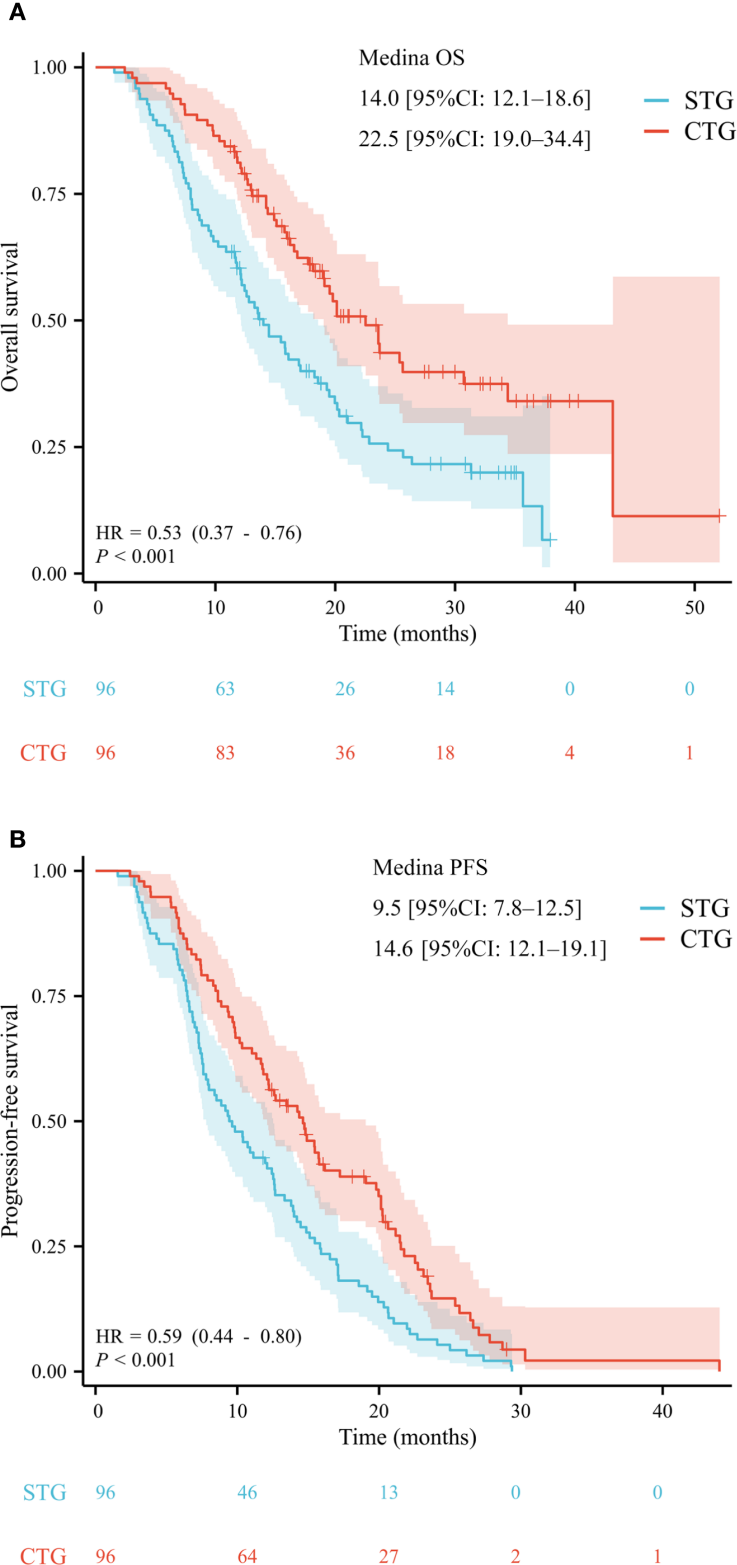
After propensity score matching, the Kaplan–Meier analysis of overall survival **(A)** and progression-free survival **(B)**.

With a median follow-up of 28.6 months (IQR 18.8–34.7), the combination therapy group demonstrated significantly superior survival outcomes compared to systemic therapy alone. After PSM, the median OS was 22.5 months (95% confidence interval [CI] 19.0–34.4) versus 14.0 months (95% CI 12.1–18.6), corresponding to a 47% reduction in mortality risk (hazard ratio [HR] 0.53, p<0.001) (**Figure 2 A**). The median PFS was significantly prolonged in the combination group (14.6 months, 95% CI 12.1–19.1 *vs*. 9.5 months, 95% CI 7.8–12.5; HR 0.64, p<0.001) (**Figure 2 B**).

### Prognostic factor analysis

Multivariate analysis of the entire cohort identified five independent predictors of poor OS: age <60 years (HR 1.503, 95% CI 1.109–2.036; p=0.008), extrahepatic spread (HR 2.114, 95% CI 1.550–2.884; p<0.001), portal vein tumor thrombus (HR 1.480, 95% CI 1.083–2.023; p=0.014), AFP ≥400 ng/mL (HR 2.317, 95% CI 1.679–3.198; p<0.001), and elevated GGT (cut-off value =107.003; per-unit increase HR 1.002, 95% CI 1.001–1.002; p<0.001). Details are shown in **Table 2**. Subgroup analysis within the combination therapy group revealed maximal survival benefit in patients aged ≥60 years (HR 0.593, 95% CI 0.397–0.886; p=0.011), without extrahepatic spread (HR 0.578, 95% CI 0.381–0.877; p=0.010), AFP <400 ng/mL (HR 0.493, 95% CI 0.326–0.746; p=0.001), and normal GGT levels (cut-off value =97.589; per-unit decrease HR 0.999, 95% CI 0.998–1.000; p=0.037). Details are shown in **Table 3**.

**Table 2:** Univariate and multivariate analysis of factors associated with overall survival.

Variables	Univariate analysis			Multivariate analysis			
	HR	95% *CI*	*P*-value	HR	95% *CI*	*P*-value	
Sex, Female	1.216	0.759,1.946	0.416				
Age≥60, (years)	0.663	0.476,0.924	0.015	0.665	0.492,0.901		0.008^*^
Surgical resection	1.079	0.753,1.546	0.678				
TKIs (*vs* Lenvatinib)							
Sorafenib	0.537	0.220,1.307	0.171				
Regorafenib	0.501	0.164,1.525	0.223				
Apatinib	0.511	0.210,1.242	0.138				
Donafenib	0.694	0.164,2.941	0.620				
TACE	0.426	0.300,0.605	<0.001	0.449	0.326,0.617		<0.001^*^
Etiology	1.526	0.969,2.403	0.068				
Extrahepatic spread	2.641	1.537,4.536	<0.001	2.114	1.550,2.884		<0.001^*^
Tumor diameter≥5cm	1.081	0.766,1.527	0.657				
Tumor number≥3	0.995	0.699,1.418	0.980				
Portal vein tumor thrombus	1.818	1.124,2.940	0.015	1.480	1.083,2.023		0.014^*^
Child-Pugh class C	1.057	0.721,1.551	0.776				
BCLC stage C	1.363	0.695,2.675	0.367				
MELD score≥18	0.853	0.546,1.332	0.485				
AFP ≥400(ng/ml)	2.365	1.704,3.282	<0.001	2.317	1.679,3.198		<0.001^*^
Platelet≥100(10^9/L)	0.896	0.724,1.109	0.314				
PT(sec)	6.100	0.486,76.612	0.161				
INR	1.001	0.969,1.034	0.953				
Albumin(g/l)	2.031	0.860,4.798	0.106				
Serum creatinine(mg/dL)	1.002	1.001,1.003	0.001				
GGT(U/L)	1.000	1.000,1.000	0.156	1.002	1.001,1.002		<0.001^*^
Cholinesterase(U/L)	0.886	0.694,1.131	0.332				
Total bilirubin(mg/dl)	1.007	0.999,1.016	0.096				
Hemoglobin(g/l)	0.755	0.530,1.075	0.119				
Lymphocyte count(10^9/L)	0.962	0.745,1.241	0.765				

**Table 3:** Univariate and multivariate analysis of factors associated with overall survival in CTG.

Variables	Univariate analysis			Multivariate analysis			
	HR	95% *CI*	*P*-value	HR	95% *CI*		*P*-value
Sex, Female	0.973	0.557,1.700	0.924				
Age≥60, (years)	0.558	0.355,.877	0.011	0.593	0.397,0.886		0.011^*^
Surgical resection	1.081	0.638,1.833	0.771				
TKIs (*vs* Lenvatinib)			0.806				
Sorafenib	0.867	0.328,2.290	0.773				
Regorafenib	1.262	0.746,2.134	0.386				
Apatinib	1.633	0.483,5.522	0.430				
Donafenib	1.445	0.441,4.737	0.543				
Etiology	1.109	0.584,2.107	0.752				
Extrahepatic spread	2.804	1.241,6.337	0.013	1.730	1.141,2.624		0.010^*^
Tumor diameter≥5cm	1.547	0.929,2.578	0.094				
Tumor number≥3	1.120	0.669,1.877	0.666				
Portal vein tumor thrombus	2.108	0.981,4.530	0.056				
Child-Pugh class C	0.892	0.538,1.480	0.658				
BCLC stage C	0.443	0.167,1.176	0.102				
MELD score≥18	1.076	0.576,2.009	0.819				
AFP ≥400(ng/ml)	2.077	1.340,3.218	0.001	2.027	1.340,3.068		0.001^*^
Platelet≥100(10^9/L)	0.957	0.700,1.308	0.782	0.621	0.409,0.942	0.025	0.621
PT(sec)	4.249	0.134,134.792	0.412				
INR	1.015	0.977,1.055	0.443				
Albumin(g/l)	1.160	0.329,4.088	0.818				
Serum creatinine(mg/dL)	1.002	1.000,1.003	0.134				
GGT(U/L)	1.000	1.000,1.000	0.314	1.001	1.000,1.002		0.037^*^
Cholinesterase(U/L)	0.851	0.599,1.209	0.367				
Total bilirubin(mg/dl)	1.005	0.993,1.018	0.405				
Hemoglobin(g/l)	0.634	0.388,1.037	0.070				
Lymphocyte count(10^9/L)	0.890	0.632,1.254	0.505				

CTG, combination therapy group; TKIs, tyrosine kinase inhibitors; BCLC, barcelona clinic liver cancer; MELD, Model for end-stage liver disease; AFP, alpha-fetoprotein; PT, prothrombin time; INR, international normalized ratio; GGT, gamma-glutamyl transferase. ^*^A P-value <0.05 was considered to indicate statistical significance.

### Safety profile

Safety analysis showed significantly higher rates of all-cause adverse events in the combination therapy group compared to systemic therapy alone (79.6% *vs*. 67.0%, p=0.021), with notably increased grade ≥3 events (23.5% *vs*. 14.1%, p=0.038). The combination group experienced predominantly liver-related toxicities, hematological abnormalities, and TACE-specific complications including post-embolization syndrome. Treatment discontinuation rates were comparable for tislelizumab (10.2% *vs*. 6.5%, p=0.285) and TKIs (32.7% *vs*. 27.0%, p=0.347). Summary of details are shown in **Table 4**. TACE-related AEs occurred in 46.5% of CTG patients, with Grade 3 events in 8.7% and one Grade 4 event (0.5%). Tislelizumab-related AEs were reported in 28.7% of CTG and 40.8% of STG patients, with Grade 3 events in 9.7% and 15.3%, respectively. TKI-related AEs occurred in 33.5% of CTG and 42.9% of STG patients, with Grade 3 events in 6.5% and 10.2%, respectively (**Supplementary Table 2**). In the STG, the most common any-grade AEs were fatigue (45.9%), pyrexia (40.8%), increased AST (35.7%), and increased ALT (32.7%). Grade 3 AEs were observed in 23.5% of patients, with no Grade 4 or 5 events (**Supplementary Table 3**). In the CTG, the most frequent any-grade AEs were increased AST (40.5%), increased ALT (37.8%), fatigue (37.8%), and abdominal pain (36.8%). Grade 3 AEs occurred in 14.1% of patients, with one Grade 4 abdominal pain event (0.5%). No Grade 5 events were reported in either group (**Supplementary Table 4**).

**Table 4:** Adverse events from all cause.

Variable	CTG(n=185)	STG(n=98)	*P*-value
Patients with an adverse event from all cause	124 (67.0%)	78 (79.6%)	0.021^*^
Grade <3 event	98 (53.0%)	55 (56.1%)	0.621
Grade ≥3 event	26 (14.1%)	23 (23.5%)	0.038^*^
Discontinuation of tislelizumab therapy	18 (9.7%)	15 (15.3%)	0.158
Discontinuation of TKIs therapy	12 (6.5%)	10 (10.2%)	0.285
Dose interruption of tislelizumab therapy	35 (18.9%)	25 (25.5%)	0.194
Dose reduction or interruption of TKIs therapy	50 (27.0%)	32 (32.7%)	0.347

CTG, combination therapy group; STG, systemic therapy group; TKIs, tyrosine kinase inhibitors. ^*^A P-value <0.05 was considered to indicate statistical significance.

CTG, combination therapy group; STG, systemic therapy group; TKIs, tyrosine kinase inhibitors; TACE, transarterial chemoembolization; BCLC, barcelona clinic liver cancer; MELD, Model for end-stage liver disease; AFP, alpha-fetoprotein; PT, prothrombin time; INR, international normalized ratio; GGT, gamma-glutamyl transferase. ^*^A P-value <0.05 was considered to indicate statistical significance.

## Discussion

First-line immune checkpoint inhibitors plus anti-angiogenic therapy achieve suboptimal objective response rates in unresectable intermediate-advanced HCC, the third leading cause of global cancer deaths, limited by high tumor heterogeneity, an immunosuppressive microenvironment, and hepatic dysfunction (8, 24–26). This study retrospectively analyzed patients with unresectable HCC receiving either triple therapy, tislelizumab combined with TKIs and TACE, or dual-agent systemic treatment, demonstrating the triple regimen significantly extends median OS and reduces mortality risk. Through multivariate adjustment and subgroup analysis, we not only validated the survival advantage of this combination but also identified key beneficiary characteristics. This study will focus the discussion on the scientific value of these findings in regulating the tumor immune microenvironment and balancing treatment toxicity, as well as their potential to drive clinical practice transformation.

The magnitude of the survival benefit observed in our study is consistent with that reported in recent landmark Phase III trials evaluating combination therapies for advanced HCC. Similarly, the TALENTTACE trial (NCT04712643), which investigated atezolizumab + bevacizumab plus TACE versus TACE alone, reported a promising HR of 0.71 for PFS. Our PFS HR of 0.59 also falls within this range of high efficacy, further underscoring the robust treatment effect of the combination therapy with tislelizumab, TKIs, and TACE. Interestingly, the ORIENT-32 trial demonstrated a significant improvement in OS with sintilimab plus a bevacizumab biosimilar compared to sorafenib (median OS: not reached *vs*. 10.4 months; HR 0.57) in a Chinese population with predominantly HBV-related HCC (25). Our results are consistent with those of the LEAP-002 trial, which tested lenvatinib plus pembrolizumab versus lenvatinib alone and did not meet its dual primary endpoints of statistically significant improvement in both OS and PFS (26). These results suggest that certain combination therapy regimens may provide significant survival benefits in advanced HCC, although their efficacy may vary across different therapeutic combinations and patient populations.

The findings of this real-world study deliver three key advances in HCC treatment. First, it establishes the large-scale clinical evaluation of the PD-1 inhibitor tislelizumab in combination with TKIs and TACE. While previous pivotal trials, such as IMbrave150, validated the efficacy of the atezolizumab-bevacizumab dual regimen (27), our findings demonstrate that this triple combination significantly prolongs median OS, with an improvement substantially surpassing outcomes reported in the existing literature. Second, we identify GGT as an independent prognostic biomarker, enabling more precise patient selection. Finally, this work provides clinical confirmation of the synergistic mechanism between TACE-induced immunogenic cell death and PD-1 inhibition, thereby validating the hypothesis proposed (20, 28, 29).

The findings hold significant clinical implications. For BCLC stage B/C patients, the triple regimen reduces mortality risk by 42% in those without extrahepatic metastases, suggesting it should be the preferred treatment for this subgroup. Given the marked benefit in patients with AFP <400 ng/mL, we recommend incorporating AFP as a mandatory decision-making biomarker (30). Although grade 3 or higher adverse events occurred in 23.5% of patients, standardized dose adjustment maintained stable discontinuation rates, proving clinical feasibility. The incidence of adverse outcomes was similar to that reported in previous studies (4, 14, 31). This apparent paradox, where patients with a better inherent prognosis gain the most from intensive therapy, likely stems from differences in underlying disease biology and tolerance. Patients with high tumor burden, including extrahepatic spread and PVTT, often have aggressive disease and may lack the physiological reserve to tolerate or respond robustly to multimodal therapy (26). This limits the absolute survival benefit even from potent regimens like CTG. Conversely, patients with more favorable characteristics, such as liver confined disease or low AFP levels, possess a longer life expectancy (32). The added efficacy of CTG thus acts on a less advanced disease state, amplifying absolute survival gain by profoundly delaying progression. This serves as an example of oncology’s window of opportunity. Notably, older age, specifically being sixty years or older, was associated with significant CTG benefit, possibly reflecting less aggressive tumor biology or better tolerance. These insights emphasize that CTG is best suited for patients with significant yet non catastrophic disease burden, in whom therapy is most likely to translate into meaningful survival extension. Further studies should validate these interactions and improve patient selection strategies. These results provide high-level evidence for updating.

Several limitations must be acknowledged. First, the retrospective design may introduce selection bias. While multivariate analysis adjusted for known confounders, unmeasured variables cannot be entirely excluded. As a non-randomized study, the attribution of causality is limited by potential unmeasured confounders, despite our use of PSM to balance measurable baseline characteristics. Second, single-center data may be influenced by regional hepatitis epidemiology, necessitating multicenter validation. Variability in TACE technical parameters could affect efficacy consistency. Third, the 28.6-month median follow-up is insufficient to assess long-term immunotherapy toxicity, particularly delayed autoimmune effects of PD-1 inhibitors. Future phase III RCTs should incorporate biomarkers like PD-L1 expression and extend follow-up beyond five years for a comprehensive risk-benefit evaluation.

## Conclusion

In conclusion, combining TACE with tislelizumab-TKIs significantly improves survival over systemic therapy alone in unresectable HCC, with maximal benefit observed in patients aged ≥60 years, without extrahepatic spread, with AFP <400 ng/mL, or normal GGT, despite increased manageable toxicity.

## Data Availability

The raw data supporting the conclusions of this article will be made available by the authors, without undue reservation.
