# Supplementary material for: Tislelizumab plus tyrosine kinase inhibitors with TACE improves survival in unresectable hepatocellular carcinoma with clinical predictors and manageable safety
Source: Front Immunol. 2025 Sep 29;16:1664519. doi: 10.3389/fimmu.2025.1664519 (PMC12515662; doi:10.3389/fimmu.2025.1664519)
Supplement: Supplementary file 1 [file DataSheet1.pdf]

Supplementary Table 1. Patient baseline characteristics after PSM

Characteristics, n (%)	CTG(n=96)	STG(n=96)	P-value
Sex			0.092
Male	79 (41.1%)	87 (45.3%)	
Female	17 (8.9%)	9 (4.7%)	
Age, (years)			0.107
<60	34 (17.7%)	45 (23.4%)	
≥60	62 (32.3%)	51 (26.6%)	
Age, median (IQR)	63 (55.7, 68)	60 (54, 66)	0.064
Surgical resection			0.561
Yes	44 (22.9%)	40 (20.8%)	
No	52 (27.1%)	56 (29.2%)	
TKIs			0.961
Lenvatinib	51 (26.6%)	48 (25%)	
Sorafenib	5 (2.6%)	5 (2.6%)	
Regorafenib	35 (18.2%)	38 (19.8%)	
Apatinib	3 (1.6%)	2 (1%)	
Donafenib	2 (1%)	3 (1.6%)	
Hepatitis B			0.545
Yes	83 (43.2%)	80 (41.7%)	
No	13 (6.8%)	16 (8.3%)	
Extrahepatic spread			0.188
Yes	36 (18.8%)	45 (23.4%)	
No	60 (31.2%)	51 (26.6%)	
Tumor diameter			0.664
<5cm	43 (22.4%)	46 (24%)	
≥5cm	53 (27.6%)	50 (26%)	
Tumor diameter cm, median (IQR)	5.6 (3.1, 8.1)	5.1 (3.1, 7.8)	0.813
Tumor number			0.647
<3	31 (16.1%)	34 (17.7%)	
≥3	65 (33.9%)	62 (32.3%)	
BCLC stage			0.557

Characteristics, n (%)	CTG(n=96)	STG(n=96)	P-value
B	37 (19.3%)	41 (21.4%)	
C	59 (30.7%)	55 (28.6%)	
Portal vein tumor thrombus			0.758
Yes	30 (15.6%)	32 (16.7%)	
NO	66 (34.4%)	64 (33.3%)	
Vp type			0.911
I	1 (1.6%)	1 (1.6%)	
II	22 (35.5%)	22 (35.5%)	
III	7 (11.3%)	9 (14.5%)	
Child-Pugh class			0.336
A	66 (34.4%)	72 (37.5%)	
B	30 (15.6%)	24 (12.5%)	
MELD score			0.303
<18	35 (18.2%)	42 (21.9%)	
≥18,	61 (31.8%)	54 (28.1%)	
AFP (ng/ml)			0.274
<400	63 (32.8%)	70 (36.5%)	
≥400	33 (17.2%)	26 (13.5%)	
Platelet (10^9/L)			0.660
<100	41 (21.4%)	38 (19.8%)	
≥100	55 (28.6%)	58 (30.2%)	
PT(sec), median (IQR)	13.5 (12.2, 14.2)	13.2(12.0, 14.2)	0.566
INR, median (IQR)	1.1 (1.1, 1.2)	1.1 (1.05, 1.21)	0.220
Albumin(g/l), median (IQR)	38.3 (35, 42.6)	39.7 (34.4, 42.9)	0.755
Serum creatinine(mg/dL), median (IQR)	0.9 (0.8, 1.0)	0.9 (0.8, 1.0)	0.626
GGT（U/L), median (IQR)	67.5 (37.2, 130.6)	61.7 (30.7, 135.1)	0.652
Cholinesterase(U/L), median (IQR)	6281 (4544.5, 8215.5)	5874.5 (4291.8, 8336.8)	0.629
Total bilirubin(mg/dl), median (IQR)	1.2 (0.9, 1.9)	1.2 (0.9, 1.7)	0.459
Hemoglobin(g/l), median (IQR)	140 (124.5, 152.25)	140 (119.8, 154.0)	0.925
Lymphocyte count(10^9/L), median (IQR)	1.4 (0.8325, 1.725)	1.3 (0. 9, 1.8)	0.913

Notes: Unless otherwise indicated, data are the number of patients or median (interquartile range), with percentages in parentheses; A P-value <0.05 was considered to indicate statistical significance. PSM propensity

score matching; CTG combination therapy group; STG systemic therapy group; IQR interquartile range; TKIs tyrosine kinase inhibitors; BCLC barcelona clinic liver cancer; MELD Model for end-stage liver disease; AFP alpha-fetoprotein; PT prothrombin time; INR international normalized ratio; GGT gamma-glutamyl transferase.

Supplementary Table 2. Treatment-related adverse events

Variable	CTG(n=185)	STG(n=98)
TACE-related event	86 (46.5)	N/A
Grade 1 or 2 event	70 (37.8)	N/A
Grade 3 event	16 (8.7)	N/A
Grade 4 event	1 (0.5)	N/A
Tislelizumab -related event	53 (28.7)	40 (40.8)
Grade 1 or 2 event	35 (18.9)	25 (25.5)
Grade 3 event	18 (9.7)	15 (15.3)
TKIs-related event	62 (33.5)	42 (42.9)
Grade 1 or 2 event	50 (27.0)	32 (32.7)
Grade 3 event	12 (6.5)	10 (10.2)

Notes: CTG combination therapy group; STG systemic therapy group; TKIs tyrosine kinase inhibitors; N/A not applicable. Data are n (%), numbers represent the highest grades assigned.

Supplementary Table 3. Adverse events in systemic therapy group (n=98)

Adverse Events	All Grades	Grades 1 or 2	Grade 3	Grade 4	Grade 5
**Any adverse event**	**78 (79.6)**	**55 (56.1)**	**23 (23.5)**	**0**	**0**
Increased AST	35 (35.7)	30 (30.6)	5 (5.1)	0	0
Abdominal pain	28 (28.6)	25 (25.5)	3 (3.1)	0	0
Increased ALT	32 (32.7)	28 (28.6)	4 (4.1)	0	0
Pyrexia	40 (40.8)	38 (38.8)	2 (2.0)	0	0
Elevated bilirubin	18 (18.4)	15 (15.3)	3 (3.1)	0	0
Hypertension	15 (15.3)	10 (10.2)	5 (5.1)	0	0
HFSR	22 (22.4)	20 (20.4)	2 (2.0)	0	0
Proteinuria	12 (12.2)	10 (10.2)	2 (2.0)	0	0
Fatigue	45 (45.9)	42 (42.9)	3 (3.1)	0	0
Vomiting	20 (20.4)	18 (18.4)	2 (2.0)	0	0
Nausea	25 (25.5)	23 (23.5)	2 (2.0)	0	0
Hypothyroidism	8 (8.2)	8 (8.2)	0	0	0
RCCEP	5 (5.1)	5 (5.1)	0	0	0
Rash	18 (18.4)	16 (16.3)	2 (2.0)	0	0
Diarrhea	15 (15.3)	13 (13.3)	2 (2.0)	0	0
Thrombocytopenia	10 (10.2)	7 (7.1)	3 (3.1)	0	0
Pruritus	12 (12.2)	12 (12.2)	0	0	0
Hepatitis	5 (5.1)	3 (3.1)	2 (2.0)	0	0
Infusion related reaction	6 (6.1)	6 (6.1)	0	0	0
Pneumonitis	2 (2.0)	1 (1.0)	1 (1.0)	0	0
Enterocolitis	2 (2.0)	1 (1.0)	1 (1.0)	0	0
Decreased WBC count	8 (8.2)	6 (6.1)	2 (2.0)	0	0
Neutropenia	7 (7.1)	5 (5.1)	2 (2.0)	0	0
Hyperglycemia	9 (9.2)	8 (8.2)	1 (1.0)	0	0
Thoracic hemorrhage	1 (1.0)	0	1 (1.0)	0	0
Alopecia	4 (4.1)	4 (4.1)	0	0	0
Glomerulonephritis	1 (1.0)	0	1 (1.0)	0	0
Hyperthyroidism	3 (3.1)	3 (3.1)	0	0	0
Hypophysis	1 (1.0)	1 (1.0)	0	0	0
Elevated serum amylase	2 (2.0)	2 (2.0)	0	0	0

AST aspartate aminotransferase, ALT alanine aminotransferase, HFSR hand-foot skin reaction, RCCEP reactive cutaneous capillary endothelial proliferation, WBC white blood cell count. Data are n (%). Simulated data for illustrative purposes only. The sum of individual events exceeds the total number of patients as many patients experienced multiple events.

Supplementary Table 4. Adverse events in combination therapy group (n=185)

Adverse Events	All Grades	Grades 1 or 2	Grade 3	Grade 4	Grade 5
**Any adverse event**	**124 (67.0)**	**98 (53.0)**	**26 (14.1)**	**1 (0.5)**	**0**
Increased AST	75 (40.5)	65 (35.1)	10 (5.4)	0	0
Abdominal pain	68 (36.8)	60 (32.4)	8 (4.3)	1 (0.5)	0
Increased ALT	70 (37.8)	62 (33.5)	8 (4.3)	0	0
Pyrexia	60 (32.4)	58 (31.4)	2 (1.1)	0	0
Elevated bilirubin	40 (21.6)	35 (18.9)	5 (2.7)	0	0
Hypertension	25 (13.5)	18 (9.7)	7 (3.8)	0	0
HFSR	30 (16.2)	27 (14.6)	3 (1.6)	0	0
Proteinuria	20 (10.8)	17 (9.2)	3 (1.6)	0	0
Fatigue	70 (37.8)	65 (35.1)	5 (2.7)	0	0
Vomiting	35 (18.9)	32 (17.3)	3 (1.6)	0	0
Nausea	45 (24.3)	42 (22.7)	3 (1.6)	0	0
Hypothyroidism	15 (8.1)	15 (8.1)	0	0	0
RCCEP	10 (5.4)	10 (5.4)	0	0	0
Rash	25 (13.5)	22 (11.9)	3 (1.6)	0	0
Diarrhea	28 (15.1)	25 (13.5)	3 (1.6)	0	0
Thrombocytopenia	22 (11.9)	17 (9.2)	5 (2.7)	0	0
Pruritus	18 (9.7)	18 (9.7)	0	0	0
Hepatitis	8 (4.3)	5 (2.7)	3 (1.6)	0	0
Infusion related reaction	12 (6.5)	12 (6.5)	0	0	0
Pneumonitis	5 (2.7)	3 (1.6)	2 (1.1)	0	0
Enterocolitis	4 (2.2)	2 (1.1)	2 (1.1)	0	0
Decreased WBC count	15 (8.1)	12 (6.5)	3 (1.6)	0	0
Neutropenia	12 (6.5)	9 (4.9)	3 (1.6)	0	0
Hyperglycemia	14 (7.6)	13 (7.0)	1 (0.5)	0	0
Thoracic hemorrhage	2 (1.1)	1 (0.5)	1 (0.5)	0	0
Alopecia	8 (4.3)	8 (4.3)	0	0	0
Glomerulonephritis	2 (1.1)	1 (0.5)	1 (0.5)	0	0
Hyperthyroidism	6 (3.2)	6 (3.2)	0	0	0
Hypophysis	3 (1.6)	3 (1.6)	0	0	0
Elevated serum amylase	5 (2.7)	5 (2.7)	0	0	0

AST aspartate aminotransferase, ALT alanine aminotransferase, HFSR hand-foot skin reaction, RCCEP reactive cutaneous capillary endothelial proliferation, WBC white blood cell count. Data are n (%). Simulated data for illustrative purposes only. The sum of individual events exceeds the total number of patients as many patients experienced multiple events.
